# MEDICARE ADVANTAGE (MA) PLAN CHARACTERISTICS ON PHYSICAL AND MENTAL HEALTH OUTCOMES OF MA BENEFICIARIES

**DOI:** 10.1093/geroni/igad104.2877

**Published:** 2023-12-21

**Authors:** Rashmita Basu

**Affiliations:** East Carolina University, Greenville, North Carolina, United States

## Abstract

With growing demands for better and cost-effective care delivery by Medicare Advantage (MA) plans, comparison of self-reported health outcomes is more important because those outcomes capture patients’ experiences and perspectives. The purpose of the study is to examine the characteristics of MA plans on Medicare beneficiaries’ physical and mental health status.

Methods: I used the Medicare Health Outcomes (HOS) survey, a random sample of Medicare beneficiaries surveyed from each MA plan with a minimum of 500 enrollees conducted by the CMS. The current study included 3 baseline cohorts from 2015 (n=195,800), 2016 (n=178,973), and 2017 (n=172,047). I used physical component summary (PCS) and mental component summary (MCS) scores as two outcomes which include various domains of physical (exercise, pain, activity limitation) and mental health (social functioning, emotional status, depression) status. Plan characteristics include plan ownership status (for-profit (FP) vs. not-for-profit (NFP), plan types (PPO vs. HMO/cost contract), and the number of plan members. I used the linear regression method with clustering at the plan contract level due to the correlation of multiple beneficiaries within the same plan.

Results: Adjusted for beneficiary level characteristics, beneficiaries in PPO plans experienced greater physical and mental health scores than HMO or cost contract plans. MA plan with less than 5k beneficiaries is associated with a lower physical health score than plans with more than 100k beneficiaries. For-profit ownership was significantly associated with lower mental health scores.

Discussion: The characteristics of MA plan insurers are important factors for beneficiaries’ enrollment decisions and quality improvement efforts.

